# Early outcomes using dedicated venous stents in the upper limb of patients with venous thoracic outlet syndrome: A single centre experience

**DOI:** 10.1186/s42155-019-0066-0

**Published:** 2019-07-18

**Authors:** Saissan Rajendran, Tommy Y. Cai, Jacky Loa, Prakash Saha, Steven Dubenec

**Affiliations:** 1 Department of Vascular Surgery, Royal Prince Alfred Hospital, 50 Missenden Road, Camperdown, NSW 2066 Australia; 2 School of Medicine, University of Sydney, Edward Ford Building (A27) Fisher Road, Sydney, NSW 2006 Australia; 3 Faculty of Medicine, University of New South Wales, Wallace Wurth Building, 18 High St, Kensington, NSW 2052 Australia; 4 Academic Department of Vascular Surgery, St Thomas’ Hospital, King’s College London, Westminster Bridge Rd, London, SE1 7EH UK

## Abstract

**Introduction:**

Surgical management of Venous Thoracic Outlet Syndrome (vTOS) is based upon resection of the first rib. The optimal method to treat any residual venous scarring however remains unclear. The purpose of this study was to evaluate a single quaternary centre’s early and mid-term outcomes following endovascular reconstruction of the axillo-subclavian vein using dedicated venous stents in patients with VTOS.

**Methodology:**

A retrospective analysis of patients at Royal Prince Alfred Hospital, who underwent upper limb deep venous stenting as an adjunct in the treatment of vTOS was performed. All patients between 2012 and 2017 were included. Stent patency was assessed with duplex ultrasonography. All re-interventions and their indications were recorded.

**Results:**

A total of 24 limbs in 21 patients (13 female, median age 44 yrs) were treated with dedicated venous stents between 2012 and 2017. All patients had resection of their first rib using a transaxillary approach. Nine patients initially presented with an acute DVT and underwent thrombolysis. In three of these patients a venous stent was placed before rib resection following completion of lysis. In the remainder, the median time for stent placement following surgery was 64 days. Median follow-up from stent insertion was 50 months. Primary, primary-assisted and secondary patency at 24 months was 55%, 95% and 100% respectively with one patient lost during follow-up. There were no major complications. A total of 14 re-interventions were performed on these patients. Three patients reported residual symptoms following stenting including heaviness (*n* = 1), bluish discolouration (*n* = 1) and prominent veins on the chest (*n* = 1) with the remainder asymptomatic.

**Conclusion:**

In this single centre study, endovascular reconstruction using dedicated venous stents appears to be an effective and safe method to reconstruct a damaged subclavian vein following rib resection in patients with vTOS.

## Introduction

Venous thoracic outlet syndrome (vTOS) occurs due to an anatomical narrowing of the costoclavicular triangle through which the axillo-subclavian vein passes. One variant of this condition is Paget–Schroetter syndrome (PSS) or effort- induced thrombosis of the subclavian vein which has an incidence of 1–2/100,000 people and was first described in 1875 (Von Schroetter 1884; Paget 1875). The narrowing of the costo-clavicular space may not be sufficient to cause venous thrombosis, but repetitive movement into provocative positions such as abduction and external rotation of the upper limb causes repeated compression-decompression of the axillo-subclavian vein. The resulting chronic microtrauma to the vessel leads to hypertrophy of the venous intima and inflammation resulting in stenosis and thrombosis of the axillo-subclavian vein (Aziz et al. 1986).

To date there are no well-conducted randomized control trials that look specifically at PSS interventions and their outcomes with the majority of evidence coming from case series. Although a rare presentation, the condition typically affects young people and can be debilitating, especially if the dominant arm is most often affected. Current clinical experience appears to suggest that without recanalizing the axillary vein and removing the precipitant anatomical defect, patients are subject to variable, but significant rates of re-thrombosis (Elman and Kahn 2006). With the inclusion of modern endovascular technologies removal of thrombus in the acute setting with catheter directed thrombolysis (CDT) is possible and it generally accepted that this may be combined with the correction of the underlying anatomical abnormality through open surgical thoracic outlet decompression (Stone et al. 2010; Vik et al. 2009; Rutherford 1998). The optimal method to treat residual venous scarring, however, remains unclear. Options include open veno-patchplasty at the time of surgical decompression and balloon angioplasty, which may be needed multiple times. With the advent of endovascular stents that have been designed for use in the venous system we have, however, attempted to reconstruct the venous system using percutaneous techniques. The purpose of this study was to evaluate a single quaternary centre’s early and mid-term outcomes following endovascular reconstruction of the axillo-subclavian vein using dedicated venous stents in patients with VTOS.

## Methods

A retrospective analysis of patients at Royal Prince Alfred Hospital, who underwent upper limb deep venous stenting as an adjunct in the treatment of vTOS was performed. All patients between 2012 and 2017 were included. Patient demographics, mode of presentation, presenting symptoms and vascular risk factors were recorded. Initial patient management and time to definitive treatment from symptoms were identified. Operative data was also recorded. Outcomes include: stent patency, re-intervention rates and change in symptom severity. Outcomes were analysed using the Kaplan–Meier method.

### Treatment algorithm

In the vascular surgery unit at Royal Prince Alfred Hospital (Sydney, Australia) there is a standardized approach to the management of PSS. Diagnosis is made based on detailed clinical history and duplex ultrasonography. All patients are commenced on a heparin infusion and if the patient has an appropriate risk-benefit profile and symptom onset suggestive of venous thrombosis within 2 weeks of presentation, we offer CDT. If there are contraindications for thrombolysis, patients are managed with long-term oral anticoagulation.

Our CDT protocol involves up to 72 h of urokinase infusion with daily check venograms to assess progress. If a short segment stenosis is recognized during venography during or after successful thrombolysis, this is angioplastied to improve the luminal diameter in order to maintain flow through the major axial vein. The patient is discharged home with oral anticoagulation or subcutaneous low molecular weight heparin, and brought back for elective first rib resection via a transaxillary approach within a month of hospital discharge.

**Fig. 1:**
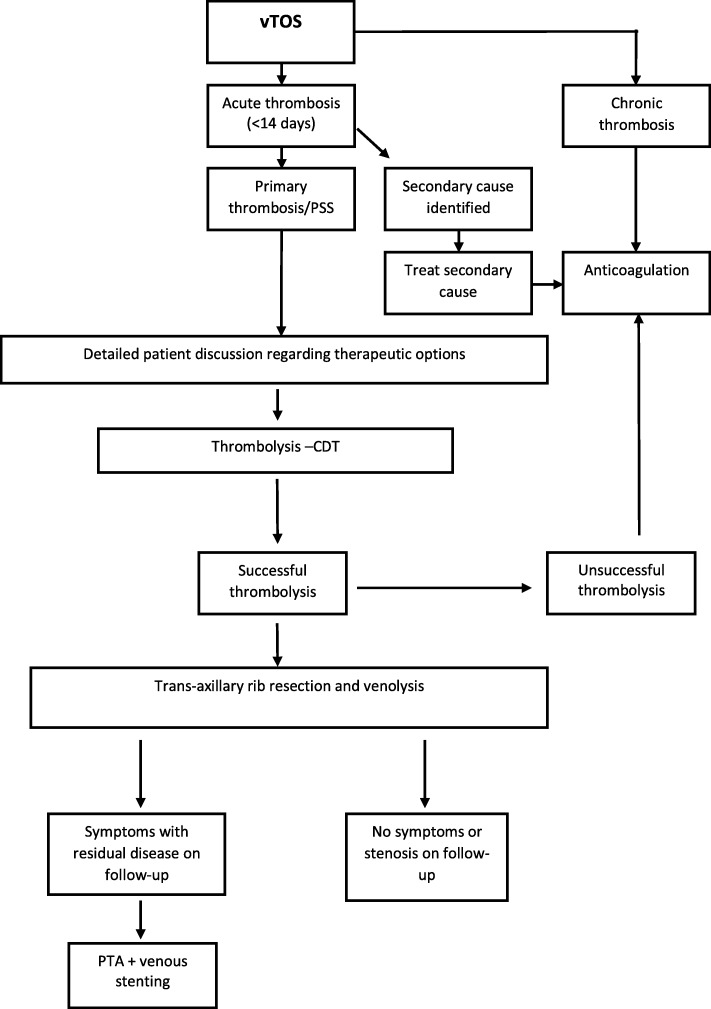
Royal Prince Alfred Hospital treatment algorithm for venous thoracic outlet syndrome. vTOS, venous thoracic outlet syndrome; CDT, catheter-directed thrombolysis; PTA, percutaneous transluminal angioplasty

Following surgical decompression of the thoracic outlet, patients typically follow-up within 1 month for clinical and ultrasonographic assessment with provocative manoeuvres to determine venous patency and residual stenosis. In symptomatic patients in whom a residual stenosis was detected, a venous stent was placed following appropriate counselling. This generally occurred within the first month of rib resection. Figure 1 summarises our treatment algorithm.

## Results

A total of 24 upper limbs in 21 patients had a venous stent placed. Of these 21 patients 13 (62%) were female. The median age was 44 (Range 21–67). Of the limbs treated 13 (54%) were right sided. Nine patients initially presented with acute upper limb DVT and underwent thrombolysis. The majority of patients presented with arm pain (13/21), swelling (14/21) and pallor (5/21). Table 1 highlights the patient demographics.

**Table 1:** Patients’ risk factors for vascular disease and thrombus formation

Median Age	44 (21–67)
Gender	13 (62%) female
Hypertension	0
Diabetes	0
Current Smoker	4
Ex-smoker	5
Thrombophilia	0
Previous Thrombosis	2
Traumatic Etiology	0

All patients had resection of their first rib using a transaxillary approach. Of the 9 patients who had presented with acute vTOS, three had a venous stent following completion of lysis and prior to rib resection. These were performed prior to the implementation of our treatment protocol (Fig. 1). In the remainder of patients, the median time for stent placement following surgery was 64 days. Table 2 highlights the stents used, which were all open cell in design.

**Table 2:** Brands of stents used in the treatment of vTOS

Stent	Number (%)
Cook® Zilver Vena™	15 (63)
Bard® Venovo™	7 (29)
Optimed® Sinus Venous™	1 (4)
Medtronic® Absolute Pro™	1 (4)

**Fig. 2:**
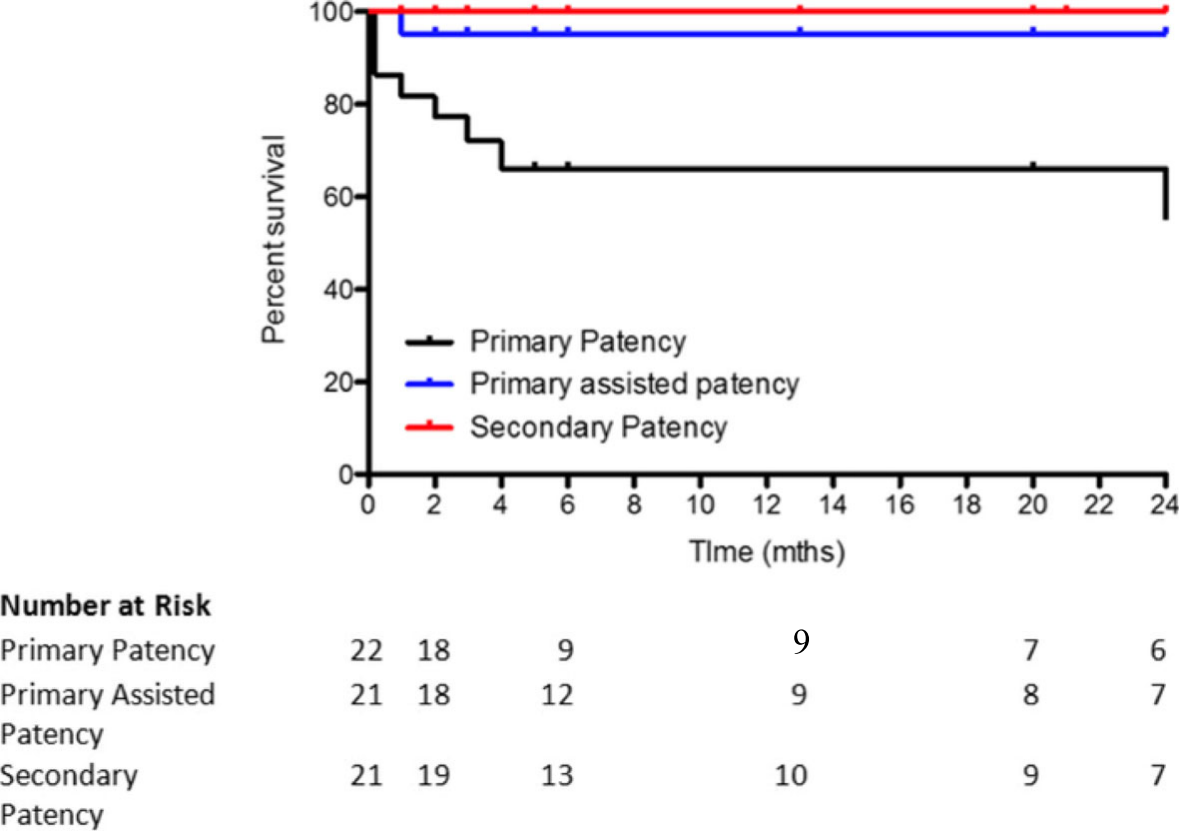
Kaplan–Meier curve for venous stent patency

Primary, primary-assisted and secondary patency at 24 months was 55%, 95% and 100% respectively (Fig. 2). Median follow-up from stent insertion was 50 months. There were no major complications. We had a total of 14 re-interventions. One person presented with acute thrombosis of their stent, which was successfully lysed and an extension stent was placed into the axillary vein. One patient presented with a fracture of their stent that was re-lined. All other re-interventions consisted of venoplasty of which 3 patients required more than one re-intervention. Of the 3 patients who had rib resections after stent insertion, two required further angioplasty following surgical decompression. Three patients reported residual symptoms following stenting including heaviness (*n* = 1), bluish discolouration (*n* = 1) and prominent veins on the chest (*n* = 1) with the remainder asymptomatic.

## Discussion

**Fig. 3:**
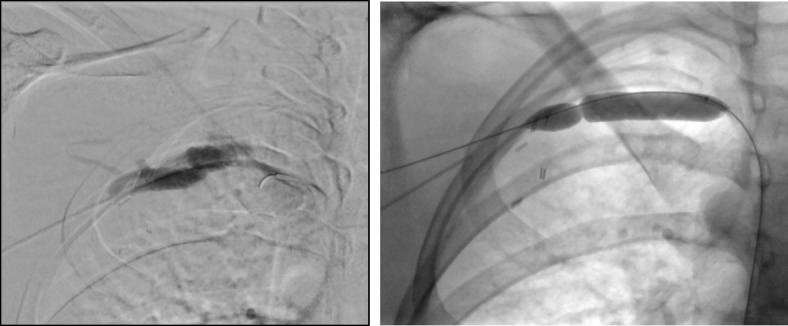
Venogram post rib resection confirming stenosis in the axillo-subclavian vein and balloon angioplasty

**Fig. 4:**
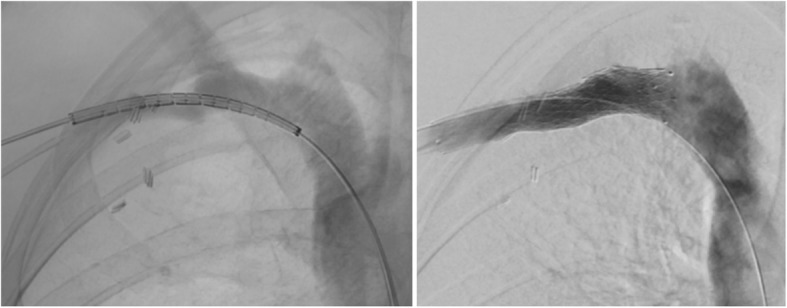
Residual stenosis post angioplasty and result post stenting

This study highlights promising early to mid-term outcomes of venous stenting in the thoracic outlet for patients that present with vTOS. As it is a condition that predominately affects a younger population, it can lead to significant morbidity in patients who are not treated affecting their long-term functional ability. Treatment is paramount when the dominant hand is affected. Treatment algorithms involving early CDT and adjuvant thoracic outlet decompression as a treatment modality is associated with significant improvements in long term outcomes (Rutherford 1998). Our results, although in a small number, suggest however that there could be a role for adjuvant therapies including follow-up venography with percutaneous transcutaneous angioplasty (PTA) and venous stenting for patients with residual stenosis on duplex ultrasonography. The residual stenosis is likely caused by endothelial fibrosis similar to that found in May-Thurner syndrome. Similar to the pelvic veins, these residual venous lesions are refractory to angioplasty alone as there is a high rate of recoil (Figs. 3 and 4). Leaving such lesions may therefore increase the risk of recurrent thrombosis despite decompression of the thoracic outlet. We therefore advocate venous stenting to be beneficial by increasing the luminal diameter and maintaining flow through diseased axial veins.

**Fig. 5:**
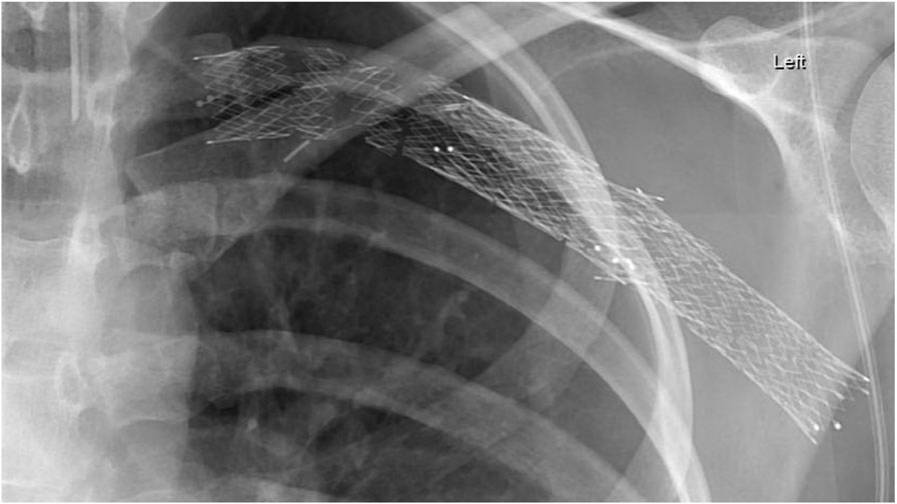
Optimed™ sinus venous stent 18 months after placement

There is some anecdotal evidence that there is an unacceptably high risk of stent fracture secondary to the dynamic compression of the stent with abduction of the arm, even after rib resection. We have seen this with one stent type as shown in Fig. 5, however, our experience with the older Cook® Zilver Vena™ and the current generation specific venous stent, the Bard® Venovo™ Venous Stent has not had this problem albeit with a short follow-up period. These open cell stents that have been designed to have crush resistance within the post-thrombotic vein while still maintain flexibility.

The ideal stent design in this region should conform to the tortuous anatomy, accommodate the free range of shoulder movement and withstand compression with a high radial force. Although there are no dedicated venous stents for the thoracic outlet, we have had much success with the current generation dedicated venous self-expanding nitinol stents. Typically, the stent sizes required are around 14 mm in diameter, accommodating the size of the axillary and subclavian vein. The length of the stent required needs to completely cover the damaged segment of vein with the same principles applied to pelvic veins applied to this anatomical area. Use of intravascular ultrasound (IVUS) here can be very beneficial as seen within the pelvic system. The use of IVUS at our institution was at the discretion of the operating surgeon. The main benefits included accurate demonstration of the diseased segment and true luminal diameters allowing appropriate length and diameter stent sizing. Treatment of this segment hinges on ensuring adequate outflow and inflow and achieving appropriate luminal cross-sectional area.

With the Cook® Zilver Vena™, stenting was possible from the arm due to the 7 french sheath required. With the newer dedicated venous stents such as the Bard® Venovo™ a 9 french sheath is required is required for access. This required a change to a femoral approach. Most cases required either an arm or femoral vein puncture depending on the stent used, however, access through the lesion from a dual approach from basilic/brachial and femoral veins was also employed when lesions were challenging to cross. This flossing technique provides a stable platform when delivering balloons and stents. As with iliac lesions, pre and post-dilatation of the vessel to the stent size was performed to ensure adequate stent opening and decent luminal diameter.

After stent placement our patients were therapeutically anticoagulated for at least 3 months using a vitamin K antagonist or a direct thrombin inhibitor to prevent thrombosis in areas where there has been prior luminal damage. Following this we recommended lifelong aspirin. We used an aggressive post-operative surveillance with ultrasound performed 6 weeks, 3 months, 6 months and 12 months post intervention provided there was no change in symptoms.

Our study has demonstrated relief of symptoms in 88% of patients during follow-up. In 1993, Machelder published an algorithm of treating vTOS with early CDT and rib resection. He demonstrated that 93% of patients with patent axillo-subclavian veins after first rib resection were symptom-free at a mean follow-up of 3.1 years and that only 64% were symptom-free if the vein had occluded (Machleder 1993). Our results are comparable to Machelder and highlights the importance of achieving venous patency with reconstruction and strict follow-up.

There are several limitations of this study including the limitations of retrospective data collection, small sample size and limited follow up time. The small numbers found in this study is not uncommon to other literature regarding vTOS. However, this lack of power makes it difficult to reach firm conclusions regarding the effectiveness of adjunctive venous thoracic outlet stenting. Multicenter, prospective trials over a longer period of time are therefore needed to fully evaluate the impact of this proposed management strategy. Although there is no evidence available for the optimal management of vTOS, our study demonstrats that our treatment algorithm involving adjunctive venous stenting should be considered in selected patients.

## Conclusion

Our early results of venous stenting have been promising. With the advent of improved venous stent technology, this is a safe procedure that avoids the complexities of open venous intervention and is more durable to endovascular venous angioplasty alone. The long-term outcomes and durability of stenting in this cohort of patients is, however, yet to be determined.

## Abbreviations

CDT: Catheter directed thrombolysis
PSS: PAGET–Schroetter syndrome
PTA: Percutaneous transcutaneous angioplasty
vTOS: Venous thoracic outlet syndrome

## Availability of data and supporting materials

Please contact author for data requests.
